# Differential growth factor regulation of aspartyl-(asparaginyl)-β-hydroxylase family genes in SH-Sy5y human neuroblastoma cells

**DOI:** 10.1186/1471-2121-7-41

**Published:** 2006-12-07

**Authors:** Stephanie A Lahousse, Jade J Carter, Xaolai J Xu, Jack R Wands, Suzanne M de la Monte

**Affiliations:** 1 Departments of Medicine and Pathology, Rhode Island Hospital, Brown Medical School, and the Pathobiology Graduate Program, Brown University, Providence, RI, USA

## Abstract

**Background:**

Aspartyl (asparaginyl)-β-hydroxylase (AAH) hydroxylates Asp and Asn residues within EGF-like domains of Notch and Jagged, which mediate cell motility and differentiation. This study examines the expression, regulation and function of AAH, and its related transcripts, Humbug and Junctin, which lack catalytic domains, using SH-Sy5y neuroblastoma cells.

**Results:**

Real time quantitative RT-PCR demonstrated 8- or 9-fold higher levels of Humbug than AAH and Junctin, and lower levels of all 3 transcripts in normal human brains compared with neuroblastic tumor cells. AAH and Humbug expression were significantly increased in response to insulin and IGF-I stimulation, and these effects were associated with increased directional motility. However, over-expression of AAH and not Humbug significantly increased motility. Treatment with chemical inhibitors of Akt, Erk MAPK, or cyclin-dependent kinase 5 (Cdk-5) significantly reduced IGF-I stimulated AAH and Humbug expression and motility relative to vehicle-treated control cells. In addition, significantly increased AAH and Humbug expression and directional motility were observed in cells co-transfected with Cdk-5 plus its p35 or p25 regulatory partner. Further studies demonstrated that activated Cdk-5 mediated its stimulatory effects on AAH through Erk MAPK and PI3 kinase.

**Conclusion:**

AAH and Humbug are over-expressed in SH-Sy5y neuroblastoma cells, and their mRNAs are regulated by insulin/IGF-1 signaling through Erk MAPK, PI3 kinase-Akt, and Cdk-5, which are known mediators of cell migration. Although AAH and Humbug share regulatory signaling pathways, AAH and not Humbug mediates directional motility in SH-Sy5y neuroblastoma cells.

## Background

Aspartyl (Asparaginyl) β-Hydroxylase (AAH) is a Type 2 transmembrane protein that has a predicted molecular mass of ~86 kD [1]. AAH is a member of the α-ketoglutarate-dependent dioxygenase family of molecules [2,3], and catalyzes the hydroxylation of specific aspartyl and asparaginyl residues in EGF-like domains of certain proteins [4,5]. The consensus sequence for AAH hydroxylation is present in Notch, Jagged, and extracellular matrix molecules such as laminin and tenascin, which have demonstrated roles in cell motility or adhesion [4,5]. The proposed AAH hydroxylation reaction uses molecular oxygen to form succinate, carbon dioxide, and 3-hydroxyaspartic acid [6]. The catalytic domain resides within the carboxyl terminus and corresponding ~52 kD cleavage product of AAH [7].

The ~200 kB AAH gene encodes 3 proteins, AAH, Humbug, and Junctin [5,8,9], which are generated by alternative splicing and exon sharing [5]. There are two AAH mRNA transcripts that encode identical proteins, which differ only in length of the 3'-untranslated region [1,5]. Humbug is derived from the first 13 exons of the AAH gene, and lacks the C-terminal region that is responsible for catalytic activity in AAH [4,5,9,10]. Junctin is the smallest of the 4 transcripts, and contains Exons 1A, 2, 3, 4A, and 5A of the AAH gene [9]. Therefore, all 3 AAH-related proteins share common N-terminal exons that encode a trans-membrane domain in addition to a portion of the cytoplasmic domain [4,9] but differ in the length and function of the C-terminus.

AAH is abundantly expressed in a broad range of malignant neoplasms and transformed cells lines, including those of hepatic, biliary, breast, intestinal, pulmonary, pancreatic, and neural origin, whereas most normal mature tissues have relatively low levels of AAH [1,11-14]. However, placenta is a notable exception in that motile and invasive trophoblasts express high levels of AAH [1,15]. Initial studies established a convincing role for AAH in malignancy by demonstrating transformation of NIH3T3 cells that were stably transfected with the human AAH cDNA, and partial reversal of the transformed phenotype in cells that were transfected with a dominant negative AAH mutant that lacked catalytic activity [11]. In situ studies demonstrated that the highest levels of AAH immunoreactivity were localized at the infiltrating margins of malignant neoplasms, rather than in their centers [1,13,14]. The peripheral distribution of prominent AAH immunoreactivity was not correlated with zonal differences in cell viability or proliferation [14], and correspondingly, proliferation states that were un-related to transformation, such as hepatocyte or bile duct regeneration, and pre-malignant conditions such as primary sclerosing cholangitis, were found to have low (normal) levels of AAH [11]. Therefore, enhanced AAH expression is not correlated with cell proliferation per se. Instead, the findings of increased AAH immunoreactivity along the infiltrating margins of tumors and in metastatic foci [1,13,14], together with the high levels of AAH in trophoblastic cells, which are normally motile and invasive, led us to hypothesize that AAH has a functional role in cell motility [14,16].

Humbug is also abundantly expressed in malignant neoplasms of diverse histogeneses, including carcinomas of hepatic, biliary, colonic, and pulmonary origin, as well as various transformed cell lines [4,5,9,17]. Humbug can bind calcium, and over-expression of Humbug results in increased intracellular levels of calcium due to its release from intracellular stores [9,10]. Thus far, Junctin expression has been characterized in skeletal and cardiac muscle [5,9], but not in malignant neoplastic cells. Like Humbug, Junctin has a role in regulating calcium release from the sarcoplasmic reticulum [4,5,9,18,19]. In addition, Junctin can physically associate with the ryanodine receptor complex, and may have an important role in stabilizing the complex [4,5,9,18,19]. Compared with AAH, less is known about the possible function and expression of Humbug and Junctin in relation to malignancy, tumor progression, and motility.

In previous studies, a role for AAH in relation to motility was demonstrated in part by the significantly reduced levels of both AAH and directional motility observed in cells that were transfected with antisense oligodeoxynucleotides that targeted the 5'end of AAH mRNA [14,16]. However, the molecular characterization of Humbug, its structural relationship to AAH, high-level expression in malignant neoplasms, and the realization that the antisense oligodeoxynucleotides used in those experiments would have also inhibited Humbug, prompted us to further examine the expression and regulation of AAH, Humbug, and Junctin, and determine if Humbug has a role in cell motility. The strategy for examining the regulation and function of AAH and related genes evolved from a series of independent experiments demonstrating that: 1) IGF-1 promotes migration of immature neuroblastic and neuroblastoma cells [20-22]; 2) IGF-I can stimulate AAH expression [17,23]; and 3) cyclin dependent kinase-5 (Cdk-5) is an important regulator of neuronal migration in the developing central nervous system (CNS) [24-28]. The present work characterizes IGF-I regulation and downstream signaling pathways through Erk MAPK, PI3 Kinase-Akt, and Cdk-5 that modulate AAH, Humbug, and Junctin expression and directional motility in SH-Sy5y human neuroblastoma cells.

## Methods

### Cell Culture

SH-Sy5y human neuroblastoma cells, and PNET1 and PNET2 human CNS derived primitive neuroectodermal tumor cells [29] were maintained in Dulbecco's modified Eagle's medium (DMEM) supplemented with 10% fetal calf serum (FCS), 4 mM L-glutamine, 5 mM glucose, and 100 μM non-essential amino acids (Gibco-BRL, Grand Island, NY). PNET1 cells are poorly differentiated and exhibit rapid rates of proliferation, while PNET2 cells can be differentiated and exhibit intact growth factor-mediated signaling, similar to normal brain neurons [29]. To examine growth factor modulation of AAH, Humbug, and Junctin expression, sub-confluent cultures were serum starved for 12 hours, then stimulated with IGF-1 (25 nM) for up to 24 hours. Parallel cultures were stimulated with insulin (50 nM) since insulin and IGF-1 share common signaling mechanisms, or nerve growth factor (NGF; 2.5 ng/ml), which has distinct signaling mechanisms, but promotes a range of neuronal functions including neurite extension and motility [30,31]. To identify the signaling pathways likely to mediate growth factor stimulated effects on AAH, Humbug, and Junctin expression, the cells were serum-starved over-night, then treated with a chemical kinase inhibitor (Table 1), followed by IGF-1 stimulation (serum-free medium) for 24 hours. The cells were analyzed for AAH, Humbug, and Junctin mRNA expression as described below. Prior to conducting these experiments, we demonstrated by in vitro kinase assay of SH-Sy5y immunoprecipitates that, under the conditions employed, the activity of each of the targeted kinases was reduced by 80% or more relative to vehicle-treated control cells (data not shown).

**Table 1:** Kinase inhibitors

**Inhibitor**	Target*	**Concentration (μM)**
PD98059	Erk MAPK	20
SB202190	p38 MAPK	10
Akt inhibitor	Akt	8
LiCl	GSK-3β	20
Roscovitine	Cdk-5	100
H-89	PKA	2

*Erk: extracellular signal-regulated kinase; MAPK: mitogen associated protein kinase; GSK: Glycogen Synthase Kinase; cdk: cyclin-dependent kinase; PKA: Protein Kinase A

### Human Brain Tissue Samples

Normal human frontal cortex tissue was obtained at postmortem examination from 6 adults and 2 infants (6 and 8 months old). In each case, the cause of death was underlying cardiac or pulmonary disease, and the post-mortem intervals were less than 12 hours. The brain tissue samples were originally collected and banked for use in research. Adjacent blocks of fresh tissue were either fixed in neutral buffered formalin or snap frozen in a dry ice-methanol bath and stored at -80°C. Fixed tissue was embedded in paraffin and histological sections stained with Luxol Fast Blue-Hematoxylin and Eosin were used to confirm the intactness of brain parenchyma. The fresh frozen tissue was used to isolate RNA and measure gene expression by real time quantitative RT-PCR. The use of human postmortem tissue in these studies was approved by the Rhode Island Hospital-Lifespan Committee on the Protection of Human Subjects Institutional Review Board.

### Real Time Quantitative RT-PCR

Real time quantitative reverse transcriptase polymerase chain reaction (RT-PCR) studies were used to measure relative mRNA abundance of AAH, Humbug, and Junctin. Ribosomal (r) 18S levels measured in the same samples in parallel reactions were used to calculate relative abundance of each mRNA transcript [32]. Total RNA was isolated from cells and brain tissues using TRIzol reagent (Invitrogen, Carlsbad, CA) according to the manufacturer's protocol. Samples containing 2 μg of RNA were reverse transcribed using the AMV First Strand cDNA synthesis kit (Roche Diagnostics Corporation, Indianapolis, IN) and random oligodeoxynucleotide primers. PCR amplifications were performed in 25 μl reactions containing reverse transcriptase products generated from 2.5 ng of template, 300 nM each of gene specific forward and reverse primer (Table 2), and 12.5 μl of 2× QuantiTect SYBR Green PCR Mix (Qiagen Inc, Valencia, CA). The amplified signals were detected continuously with the BIO-RAD iCycler iQ Multi-Color Real time PCR Detection System (Bio-Rad, Hercules, CA). The amplification protocol was as follows: initial 10-minute denaturation and enzyme activation at 95°C, followed by 40 cycles of 95°C × 15 sec, 55°–60°C × 30 sec, and 72°C × 30 sec. Annealing temperatures were optimized using the temperature gradient program provided with the iCycler software.

**Table 2:** Real time PCR primer pairs

Primer*	**Sequence (5'→3')**	**Amplicon Size (bp)**
18 S For	GGACCAGAGCGAAAGCATTTGCC	
18 S Rev	TCAATCTCGGGTGGCTGAACGC	50
AAH For	GGGAGATTTTATTTCCACCTGGG	
AAH Rev	CCTTTGGCTTTATCCATCACTGC	256
Humbug For	GCTGGGTTGATTGAGGATGTGTG	
Humbug Rev	GCAGGGGGAAAAAGTCACCTTATC	301
Junctin For	CCTGAGTCAAGGAAGGAAAGTAAG	
Junctin Rev	GCCGTTTCTTTTCTGGGTATTTCC	308
IRS-1 For	TGCTGGGGGTTTGGAGAATG	
IRS-1 Rev	GGCACTGTTTGAAGTCCTTGACC	68
IRS-2 For	AAAATTGGCGGAGCAAGGC	
IRS-2 Rev	ATGTTCAGGCAGCAGTCGAGAG	64
IRS-4 For	CCGACACCTCATTGCTCTTTTC	
IRS-4 Rev	TTTCCTGCTCCGACTCGTTCTC	74

*For-forward; Rev-reverse; AAH = aspartyl (asparaginyl)-β-hydroxylase; IRS = insulin receptor substrate

AAH, Humbug, Junctin, and 18S RNA transcripts were simultaneously evaluated in parallel reactions using aliquots of the same cDNA templates [32]. Serial dilutions of known quantities of recombinant plasmid DNA containing AAH, Humbug, Junctin, or 18S cDNA target sequences were used as standards in the PCR reactions, and the regression lines generated from the C_t _values of the standards were used to calculate mRNA abundance. The results were normalized to 18S because 18S rRNA is highly abundant and essentially invariant, whereas housekeeping gene expression frequently varies with growth factor stimulation or treatment with kinase modulators. Inter-group statistical comparisons were made using the calculated ng ratios of AAH/18S, Humbug/18S, and Junctin/18S. In preliminary studies, the SYBR Green-labeled PCR products were evaluated by agarose gel electrophoresis, and the authenticity of each amplicon was verified by nucleic acid sequencing.

### Western Blot Analysis

Cell homogenates were prepared in radio-immunoprecipitation assay (RIPA) buffer (50 mM Tris-HCl, pH 7.5, 1% NP-40, 0.25% Na-deoxycholate, 150 mM NaCl, 1 mM EDTA, 2 mM EGTA) containing protease (1 mM PMSF, 0.1 mM TPCK, 1 μg/ml aprotinin, 1 μg/ml pepstatin A, 0.5 μg/ml leupeptin, 1 mM NaF, 1 mM Na_4_P_2_O_7_) and phosphatase (2 mM Na_3_VO_4_) inhibitors. Protein concentrations were determined using the bicinchoninic acid (BCA) assay (Pierce, Rockford, IL). Samples containing 60 μg of protein were fractionated by sodium dodecyl sulfate, polyacrylamide gel electrophoresis (SDS-PAGE) [33]. The proteins were transferred to Immobilon-P (Millipore Corporation, Bedford, MA) PVDF membranes and non-specific binding sites were adsorbed with SuperBlock-TBS (Pierce, Rockford, IL). The membranes were then incubated over night at 4°C with primary antibody (1 μg/ml) diluted in Tris-buffered saline (TBS; 50 mM Tris, 150 mM NaCl, pH 7.4) containing 1% bovine serum albumin and 0.05% Tween-20 (TBST-BSA). Immunoreactivity was detected using horseradish peroxidase (HRP) conjugated IgG (Pierce, Rockford, IL), Western Lightning chemiluminescence reagents (Perkin Elmer Life Sciences Inc., Boston, MA), and digital imaging with the Kodak Digital Science Imaging Station (NEN Life Sciences, Boston, MA).

### Microtiter Immunocytochemical ELISA (MICE) assay

The MICE assay is a rapid and sensitive method of quantifying immunoreactivity in 96-well micro-cultures [34]. The cells were fixed for 24 hours in Histochoice (Amresco, Solon, Ohio), permeabilized with 0.05% saponin in Tris-buffered saline (50 mM Tris, pH 7.5, 0.9% NaCl; TBS), and blocked with SuperBlock-TBS (Pierce, Rockford, IL). The cells were incubated overnight at 4°C with primary antibody diluted in TBS containing 0.05% Tween-20 and 0.5% bovine serum albumin (TBST-BSA). Immunoreactivity was detected with horseradish peroxidase conjugated secondary antibody (Pierce, Rockford, IL) and the TMB soluble peroxidase substrate (Pierce, Rockford, IL). Absorbances were measured at 450 nm using a Spectracount plate reader (Packard Instrument Co., Meriden, CT).

To compare the levels of protein expression it was necessary to correct for differences in cell density. After measuring immunoreactivity, the plates were washed in TBS and the cells were stained 0.1% Coomassie blue dye in 40% methanol/10% acetic acid. After extensive washing in water, the plates were dried and then the dye was eluted from the adherent cells with PBS containing 1% SDS (200 μl/well). The absorbances (560 nm) were measured using a Spectracount plate reader (Packard Instrument Company, Meriden, CT). The MICE index was calculated from the ratio of the absorbances measured for immunoreactivity and cell density multiplied by 100. Coomassie blue absorbance also increases linearly with cell density between 1 × 10^4 ^and 5 × 10^5 ^cells per well. At least 8 independent replicate cultures were analyzed in each experiment, and all experiments were repeated 3 times.

### Transfection of SH-Sy5y Cells

The full-length human AAH cDNA was ligated into the pcDNA5/FRT/TO vector (Invitrogen Corporation, Carlsbad, CA), in which gene expression was regulated by a CMV promoter (pAAH). Humbug was sub-cloned from the AAH cDNA by PCR amplification using the following primer pairs: Forward: 5'-CGG GAT CCA TGG CCC AGC GTA-3', Reverse: 5'-GCC TCG AGC CCC TTT TAC GGA G-3'. The Humbug PCR product was gel purified and ligated into the pCR3.1 mammalian expression vector (pHMBG; Invitrogen Corporation, Carlsbad, CA) in which gene expression is under the control of a CMV promoter. Orientation and authenticity of the cloned PCR product were verified by sequencing and transient transfection studies. As control, cells were transfected with recombinant plasmid expressing the luciferase gene (pLuc) that was ligated into the pcDNA3.1 vector (Invitrogen Corporation, Carlsbad, CA) in which gene expression was regulated by a CMV promoter.

To examine the effects of AAH or Humbug over-expression on directional motility, parallel cultures seeded into 35 mm^2 ^wells with 10^5 ^cells/well were transiently transfected with 4 μg plasmid DNA expressing AAH (pAAH), Humbug (pHMBG), or luciferase (pLuc; negative control), using Lipofectamine 2000 (Invitrogen Corporation, Carlsbad, CA) according to the manufacturer's protocol. To evaluate the role of Cdk-5 in relation to AAH, Humbug, and Junctin expression and motility, SH-Sy5y cells were transiently transfected with recombinant plasmids expressing Cdk-5, its regulatory partners, p25 or p35, Cdk-5+p25, or Cdk-5+p35, each of which was under the control of a CMV promoter. Cells were transfected with 2 μg of each recombinant plasmid. However, to over-express a single gene, cells were co-transfected with 2 μg recombinant plasmid + 2 μg empty vector. The use of Lipofectamine 2000 resulted in transfection efficiencies of 50%-60% in SH-Sy5y cultures, as demonstrated by co-transfection with a green fluorescent protein (GFP) reporter construct and fluorescence microscopy. In addition, transfection efficiency, time course, and peak period of gene expression were determined by luciferase assay (Promega, Madison, WI) of cells co-transfected with equivalent amounts of pLuc (0.5 μg). Finally, studies were performed to demonstrate that trypsinization and re-seeding of transiently transfected cells into fresh chambers did not significantly alter the course of transgene expression (luciferase activity) (data not shown), indicating that transiently transfected cells could be used in directional motility assays.

### Motility Assay

Directional motility was measured using the ATP Luminescence-Based Motility/Invasion (ALMI) assay [35]. Briefly, 200 μl of medium containing 25 nM IGF-1 was placed in the bottom of each blind well chamber (Neuro Probe, Gaithersburg, MD). An 8 μM pore diameter polycarbonate membrane was seated just above the trophic factor-containing medium and tightly fixed in place with the screw cap that formed the upper chamber. 100,000 viable cells (demonstrated by Trypan Blue exclusion) were seeded into the upper chamber in serum-free medium. Migration was allowed to proceed for 30 minutes at 37°C in a conventional CO_2 _incubator. The ATPLite assay (Packard Instrument Company, Meriden, CT) was used to quantify the number of cells remaining on upper surface of the membrane (non-migrated), located on the under-surface of the membrane (migrated, adherent), or distributed in the lower chamber (migrated, non-adherent) since the ATP levels are linearly correlated with cell number between 10^3 ^and 5 × 10^5 ^cells [35].

To measure ATP luminescence, non-motile cells were removed from the upper surface of the membrane using a cotton swab. The cells were lysed by immediately submerging the swabs in 200 μl of diluted ATP lysis solution in a well of a black 96-well microplate. Completeness of cell harvesting was monitored microscopically. Cells adherent to the undersurface of the membrane were harvested and lysed by submerging the wiped membrane in 200 μl of diluted ATP lysis solution in a second well of a black microplate. Cells in the lower chamber were resuspended and added directly to 25 μl of undiluted ATP lysis solution in a third well of a black microplate. After 5-minutes incubation with agitation to ensure complete cell lysis, ATPLite substrate (25 μl) was added to each well. The reactions were mixed for 2 minutes by gentle platform agitation. Subsequently, the plates were dark adapted for 5 minutes and then luminescence was measured in a TopCount Microplate reader (Packard Instrument Company, Meriden, CT). The percentages of non-motile, motile adherent, motile non-adherent cells were calculated for each assay. Experiments were performed in replicates of 6 or 8 per variable using independent cultures.

### Source of Reagents

Mouse monoclonal antibodies to AAH were generated against recombinant protein [1]. All kinase inhibitors were purchased from CalBiochem (Carlsbad, CA). The recombinant plasmids encoding Cdk-5, p35, and p25 were generously provided by Dr. Li-Hue Tsai at Harvard Medical School, Boston, MA.

### Statistical Analysis

Data depicted in the graphs represent the means ± S.D.'s of results obtained from 3 to 8 independent replicate assays. Inter-group statistical comparisons were made using Analysis of Variance (ANOVA) and the Fisher Least Significant Difference (LSD) post-hoc test with the Number Cruncher Statistical Systems, Version 2004 (Dr. Jerry L. Hintze, Kaysville, UT).

## Results

### Profiles of AAH, Humbug, and Junctin Expression in SH-Sy5y Neuroblastoma Cells

**Figure 1:**
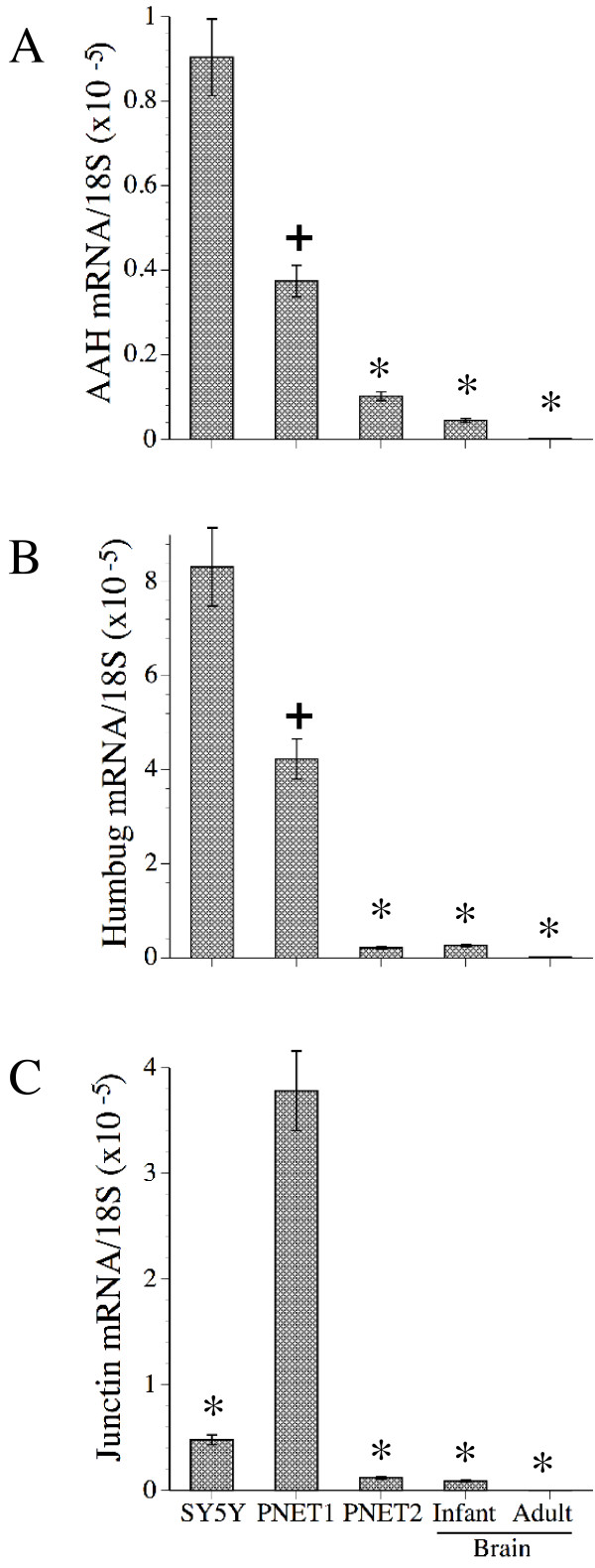
Comparison of AAH (A), Humbug (B), and Junctin (C) mRNA levels in SH-Sy5y, CNS-derived PNET1 and PNET2 primitive neuroblastic cells, normal human infant frontal cortex, and normal human adult frontal cortex. RNA was reverse transcribed with random oligodeoxynucleotide primers, and the cDNA templates were used to measure gene expression by real time quantitative PCR. The results were normalized to 18S measured in the same samples. The graphs depict the mean ± S.D. of results obtained from 6 replicate independent cultures, 2 infant brains, and 6 adult brains. Inter-group comparisons were made using ANOVA with post-hoc Fisher Least Significant Difference (LSD) tests (+ P < 0.05; * P < 0.001 relative to Sh-Sy5y cells in A and B, and relative to PNET1 cells in C).

Real time quantitative RT-PCR studies were used to examine the expression profiles and levels of AAH, Humbug, and Junctin mRNA in SH-Sy5y cells. In addition, human CNS-derived primitive neuroectodermal tumor cells (PNET1 and PNET2) and normal infant (N = 2) and adult (N = 6) human postmortem brain tissue (frontal cortex) were studied. Humbug was expressed at significantly (8 or 9 fold) higher levels than AAH or Junctin in SH-Sy5y cells (P < 0.001). In addition, the levels of AAH and Humbug mRNA were significantly higher in SH-Sy5y cells compared with PNET cells and normal human brain tissue (Figures 1A and 1B). In PNET1 cells, Humbug and Junctin mRNA transcripts were similarly abundant and both were more highly expressed than AAH (P < 0.001) (Figure 1). PNET1 cells are poorly differentiated and proliferate more rapidly compared with PNET2 cells [29]. PNET1 and PNET2 cells are less differentiated than normal infant brains, and infant brains are less differentiated than adult brains. Correspondingly, the highest levels of AAH, Humbug, and Junctin were measured in PNET1 followed by PNET2 cells, infant brain, and then adult brain (Figures 1A–1C). Moreover, in 4 of the 6 adult brain samples, AAH mRNA transcripts were not detected. It is noteworthy that the mean levels of 18S rRNA were similar in all groups (data not shown).

### Insulin/IGF-1 stimulation of AAH expression and motility

**Figure 2:**
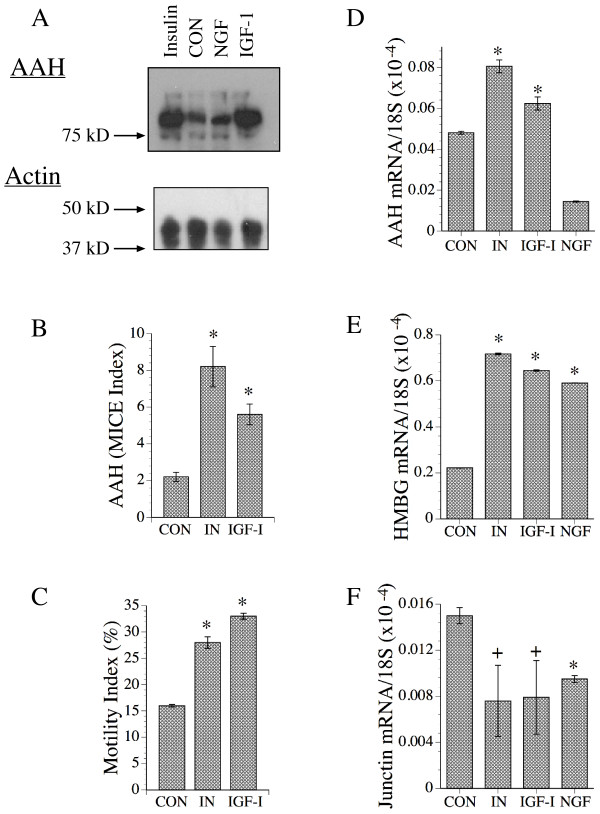
Modulation of AAH, Humbug, and Junctin expression by growth factor stimulation. Subconfluent SH-Sy5y cell cultures were serum starved over night then stimulated with vehicle (CON), insulin (50 nM), IGF-1 (25 nM), or NGF (2.5 ng/ml) for 24 hours. (A) Upper panel: Representative Western blot demonstrating AAH protein expression (~86 kD) using the HBOH monoclonal antibody. Immunoreactivity was detected with horseradish peroxidase conjugated secondary antibody, ECL reagents, and digital imaging. Lower panel-blots were stripped and re-probed to detect β-actin as a loading control. The positions of molecular weight standards included in the analysis are indicated at the left. (B) AAH immunoreactivity was measured directly in 96-well micro-cultures using the microtiter immunocytochemical ELISA (MICE) assay. The MICE index corresponds to immunoreactivity corrected for cell density. (C) Directional motility was measured in response to insulin or IGF-I stimulation using the ATP Luminescence-Based Motility/Invasion (ALMI) assay. The total percentages of motile cells (motility index), both adherent and non-adherent, were calculated (see Methods). (D-F) AAH, Humbug, and Junctin mRNA levels were measured by real time quantitative RT-PCR with results normalized to 18S. See Methods section for detailed protocols. Graphs depict mean ± S.D. of results obtained from 6 or 8 replicate independent cultures. Data were analyzed using ANOVA with the Fisher Least Significant Difference post-hoc test (+P < 0.05; *P < 0.001 relative to control).

Sub-confluent cultures of SH-Sy5y cells were serum-starved over night, then stimulated with insulin, IGF-1, NGF, or vehicle in serum-free medium for 24 hours. NGF was used as a control because it utilizes signaling mechanisms distinct from those of insulin and IGF-1, and promotes neuronal differentiation [31,36]. AAH protein expression was examined by Western blot analysis and the microtiter immunocytochemical ELISA (MICE) assay, directional motility was measured using the ATP Luminescence-based Motility/Invasion (ALMI) assay, and AAH, Humbug, and Junctin mRNA levels were measured by real time quantitative RT-PCR. Western blot analysis using the HBOH monoclonal antibody, which binds to a carboxyl terminal epitope present in AAH and not Humbug, demonstrated higher levels of AAH (~86 kD) in insulin and IGF-1-stimulated relative to NGF-stimulated and vehicle-treated control cultures (Figure 2A, upper panel). Equal loading of protein was demonstrated by probing the blots with antibodies to β-actin (Figure 2A, lower panel). Using the MICE assay which measures immunoreactivity directly in cultured cells with results normalized to culture cell density, we demonstrated significantly higher mean levels of AAH protein in insulin- and IGF-I-stimulated relative to un-stimulated cells (Figure 2B).

Directional motility was measured using the ALMI assay in which SH-Sy5y cells stimulated with vehicle, insulin, or IGF-I for 30 minutes and the mean total percentages of motile (motile-adherent + motile-non-adherent) cells were determined. The results demonstrated significantly higher mean directional motility indices in insulin- (P < 0.001) and IGF-1-stimulated (P < 0.001) relative to un-stimulated control cells (Figure 2C). In addition, IGF-I stimulated cells had a higher mean motility index than insulin-stimulated cells (P < 0.05) due to a further increase in the percentage of motile-adherent cells (migrated through the pores but remaining adherent to the membrane) within the population (data not shown).

We next compared the effects of insulin and IGF-I stimulation on AAH, Humbug and Junctin mRNA expression in SH-Sy5y cells by real time quantitative RT-PCR. As controls, parallel cultures were either treated with vehicle or NGF. Corresponding with results obtained by Western blot analysis or the MICE assay, the mean levels of AAH mRNA were significantly higher in the insulin- and IGF-1-stimulated relative to vehicle treated and NGF-stimulated cells (P < 0.001; Figure 2D). Humbug mRNA levels were significantly increased in response to IGF-1, insulin, and NGF relative to no growth factor treatment (P < 0.001; Figure 2E). In contrast, Junctin mRNA levels were not stimulated and instead were relatively suppressed with growth factor stimulation (Figure 2F).

### Potential Roles of AAH and Humbug in Relation to SH-Sy5y Cell Motility

**Figure 3:**
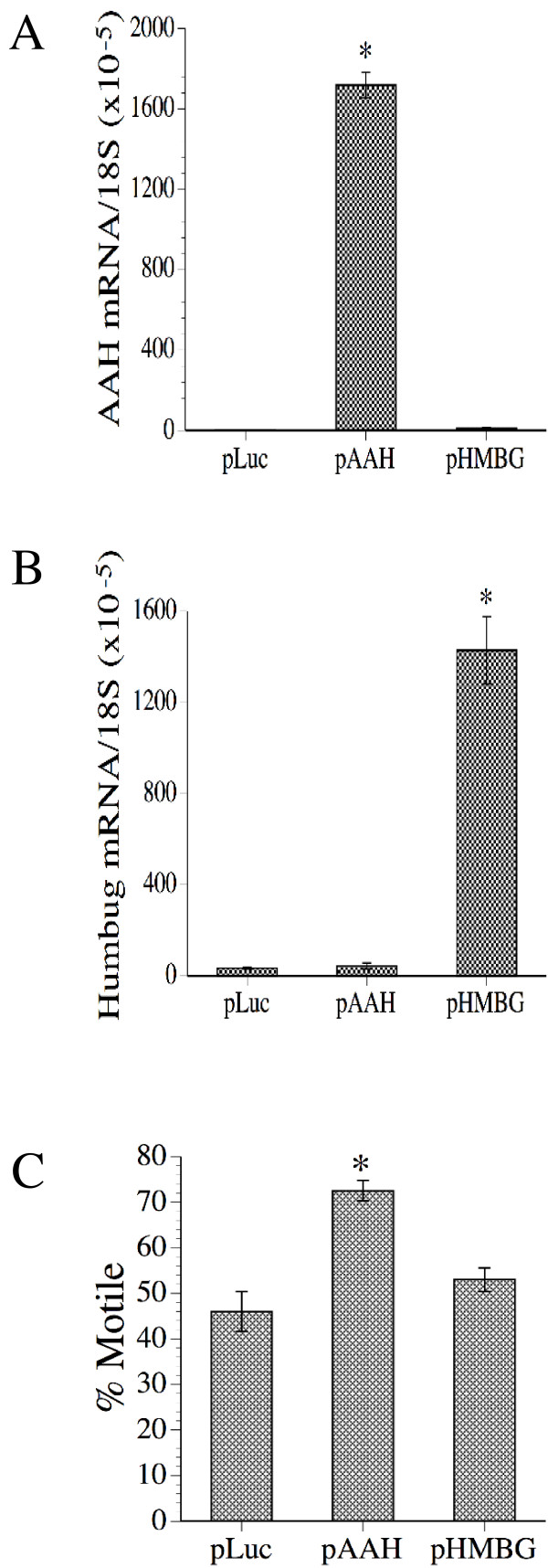
Over-expression of AAH and not Humbug increases motility in SH-Sy5y cells. SH-Sy5y cells were transfected with recombinant plasmid DNA to express luciferase (pLuc), AAH (pAAH), or Humbug (pHMBG). 48 hours after transfection during the peak period of gene expression, the cells were harvested to measure (A) AAH and (B) Humbug expression by real time quantitative RT-PCR with levels were normalized to 18S, and (C) directional motility using the ATPLite luminescence-based motility/invasion (ALMI) assay (see Methods). For the ALMI assays, percent motile refers to the combined mean percentage of motile adherent+motile non-adherent populations. The graphs depict the mean ± S.D. of results obtained from 6 or 8 replicate independent cultures. Within group comparisons were made using ANOVA with post-hoc Fisher Least Significant Difference (LSD) tests (* P < 0.001 relative to the other groups studied).

In previous studies, transfection with antisense oligodeoxynucleotides that targeted the 5' region of AAH mRNA significantly inhibited both AAH expression and motility [14,16]. However, with the additional data generated regarding the expression profiles of AAH and Humbug, it was important to determine if AAH, Humbug, or both have critical roles in regulating motility in SH-Sy5y cells. To conduct these experiments, SH-Sy5y cells were transiently transfected with cDNAs encoding AAH (pAAH), humbug (pHMBG), or luciferase (pLuc) in which gene expression was under the control of a CMV promoter. Co-transfection of pAAH and pHMBG with pLuc demonstrated equal transfection efficiencies among the groups (data not shown). Selectively increased transgene expression was verified by real time RT-PCR (Figures 3A–3B). Directional motility measured 48 hours after transfection using the ALMI assay demonstrated significantly higher mean motility indices in cells transfected with pAAH relative to those transfected with pHMBG or pLuc (P < 0.005), and similar mean motility indices in cells transfected with pHMBG or pLuc (Figure 3C).

### Pathways Mediating Insulin/IGF-1 Stimulated AAH and Humbug Expression

Results shown in Figure 2 demonstrate that AAH and Humbug expression are modulated by insulin and IGF-1 stimulation. Insulin and IGF-1 mediate their effects by activating complex intracellular signaling pathways that are initiated by ligand binding to cell surface receptors and attendant activation of intrinsic receptor tyrosine kinases [37]. Insulin/IGF-1 receptor tyrosine kinases phosphorylate insulin receptor substrates (IRS) molecules [38], which transmit signals downstream through Erk MAPK and PI3 kinase/Akt and inhibiting glycogen synthase kinase-3β (GSK-3β) to promote growth, survival, and motility [39-46].

Initial studies characterized the signaling pathways that are likely to mediate insulin and IGF-1 signaling in SH-Sy5y neuroblastoma cells. Real time quantitative RT-PCR was used to determine which IRS proteins are most abundantly expressed and how their expression levels are modulated with growth factor stimulation, and Western blot-immunoprecipitation studies were used to examine insulin/IGF-1 stimulated IRS-associated PI3 kinase activity. The real time quantitative RT-PCR studies using cells maintained in medium containing 5% FCS demonstrated that IRS-1 was the most abundantly expressed, followed by IRS-4, while IRS-2 was the least abundant of the IRS molecules expressed in SH-Sy5y cells (Table 3). IRS-3 was not studied because expression of this mRNA species is restricted to rodent adipose tissue [47]. In cultures that were stimulated with insulin or IGF-1 for 24 hours, IRS-1 mRNA levels were still highest followed by IRS-4, but IRS-1 was more abundantly expressed with IGF-1 stimulation, whereas IRS-2 and IRS-4 were more abundant in the insulin stimulated cells (Table 3). Corresponding with the real time RT-PCR results, IRS-1, tyrosyl-phosphorylated IRS-1, and p85-associated IRS-1 immunoreactivity were detected by immunoprecipitation and Western blot analysis in both insulin and IGF-1 stimulated cells, whereas IRS-2 and IRS-4 were difficult to detect (data not shown). These findings suggest that insulin and IGF-1 signaling are mainly transmitted through IRS-1 as opposed to IRS-2 or IRS-4 in SH-Sy5y cells.

**Table 3:** Levels of Insulin Receptor Substrate Expression in SH-Sy5y Cells

Growth Factor	IRS-1/18S × 10^-6^	IRS-2/18S × 10^-6^	IRS-4/18S × 10^-6^
Control	7.18 ± 0.88*, ^+^	1.19 ± 0.21	1.37 ± 0.27
Insulin	18.66 ± 2.21*, ++	2.18 ± 0.44 +	3.96 ± 0.61 +
IGF-1	32.61 ± 2.55*, ++	1.37 ± 0.24	2.01 ± 0.21 +

SH-Sy5y cells were stimulated with vehicle (Control), 50 nM insulin, or 25 nM IGF-1 for 24 hours, then analyzed by real time quantitative RT-PCR to measure mRNA levels of insulin receptor substrate (IRS) subtypes 1, 2, and 4 using the primer pairs listed in Table 2. The results were normalized to 18S ribosomal RNA measured in the same samples. The values listed represent mean ± S.D. of results obtained from 6 replicate cultures. *P < 0.001 relative to IRS-2 and IRS-4 measured in the same cultures. ^++ ^P < 0.001; ^+ ^P < 0.05 relative to control.

**Figure 4:**
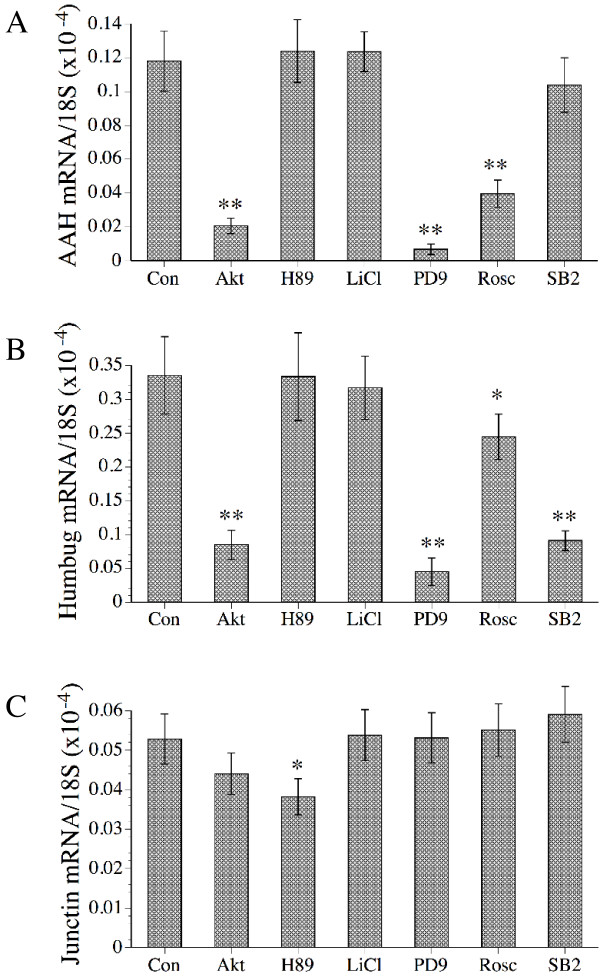
Effects of chemical kinase inhibitors on (A) AAH, (B) Humbug, and (C) Junctin mRNA levels in IGF-I stimulated SH-Sy5y cells. Subconfluent cultures were serum starved over night, then stimulated with IGF-1 and treated with vehicle (Con), or a chemical inhibitor of Akt, PKA (H-89), GSK-3β (LiCl), Erk MAPK (PD98059-PD9), Cdk-5 (Roscovitine-Rosc), or p38 MAPK (SB202190-SB2). Inhibitor concentrations are listed in Table 1. After 24 hours of growth factor stimulation, cells were harvested to measure (A) AAH, (B) Humbug, and (C) Junctin expression by real time quantitative RT-PCR as described in the legend for Figure 1. The mRNA levels were normalized to 18S rRNA levels measured in the same samples, and the graphs depict the mean ± S.D. of results. Statistical comparisons were made using ANOVA and post hoc Fisher LSD tests (**P < 0.001; *P < 0.01 relative to control).

To characterize the downstream signaling pathways that modulate AAH and Humbug expression, real time quantitative RT-PCR was used to measure AAH, Humbug, and Junctin mRNA levels in cells that were stimulated with insulin or IGF-1 and pre-treated with a chemical inhibitor of Erk MAPK (PD98059), p38 MAPK (SB202190), Akt (Akt inhibitor), GSK-3β (LiCl), cyclin dependent kinase 5 (Cdk-5; Roscovitine), or protein kinase A (H-89) (Table 1). The control cells were treated with vehicle. Studies of Cdk-5 were included because Cdk-5 is abundantly expressed in neurons and has a probable role in neuronal migration during development [26-28,48]. Since the results for insulin and IGF-1 with respect to AAH expression were similar, only data generated with IGF-1 stimulated cells are illustrated (Figure 4). The studies demonstrated significantly reduced levels of both AAH and Humbug mRNA in cells that were treated with PD98059, Akt inhibitor, or Roscovitine (P < 0.001), which inhibit Erk MAPK, Akt, and Cdk-5, respectively (Figures 4A and 4B). In addition, Humbug, but not AAH mRNA expression, was significantly reduced by treatment with SB202190, which inhibits p38 MAPK. Neither AAH nor Humbug mRNA levels were significantly modulated by treatment with H-89 (PKA inhibitor) or LiCl (GSK-3β inhibitor). Junctin mRNA transcripts were not significantly modulated by any of the kinase inhibitors, with the exception of H-89, which reduced the mean mRNA level by 40% relative to vehicle treated control cells (P < 0.05; Figure 4C).

### Effects of Erk MAPK, Akt, PI3 Kinase, or Cdk-5 Inhibition on AAH Protein Expression and Directional Motility in SH-Sy5y Cells

**Figure 5:**
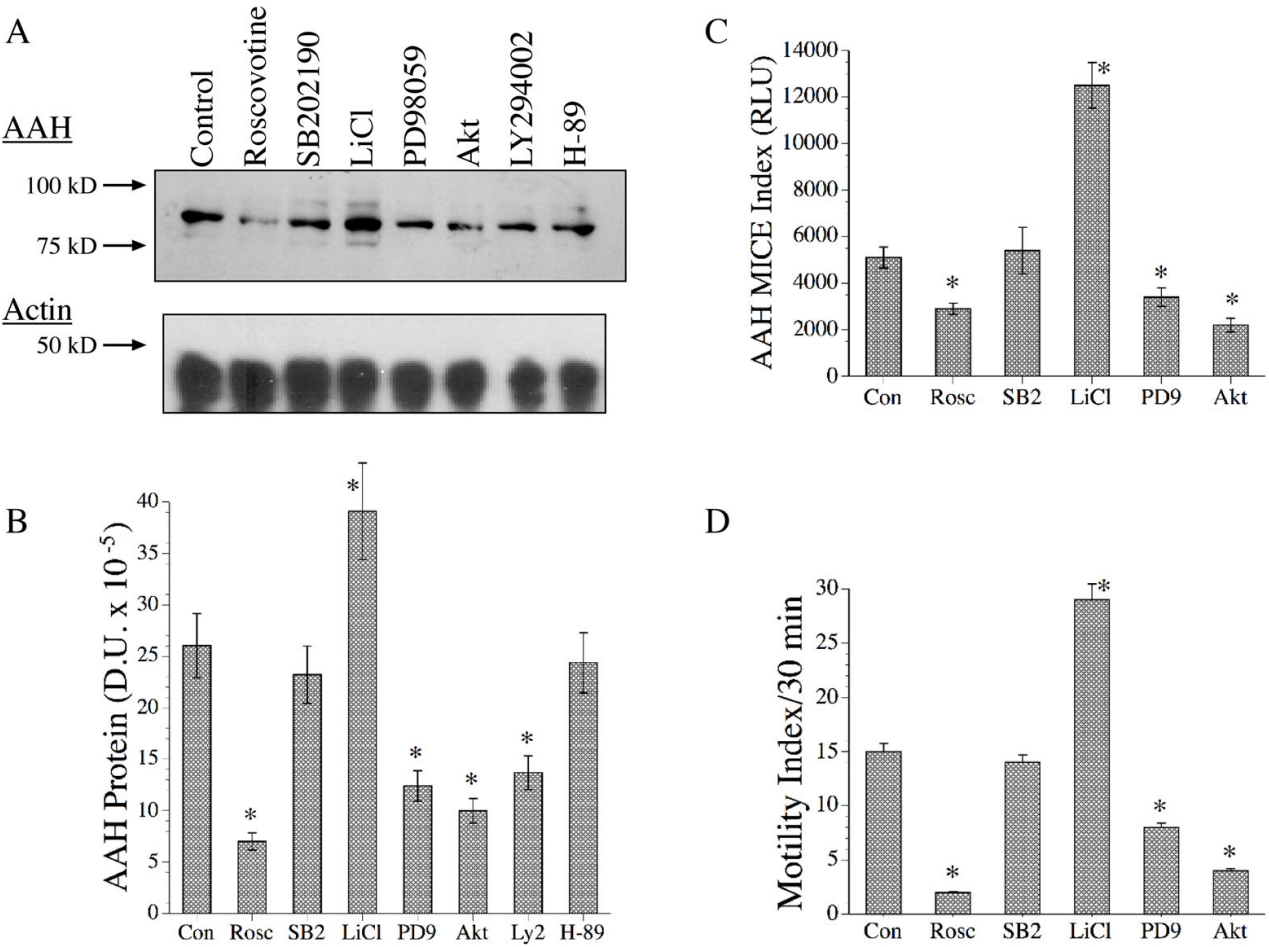
Effects of chemical kinase inhibitors on AAH protein expression and directional motility in IGF-I stimulated SH-Sy5y cells. Subconfluent cultures were serum starved over night, then stimulated with IGF-1 and treated with vehicle (Con), or a chemical inhibitor of Akt, PKA (H-89), GSK-3β (LiCl), Erk MAPK (PD98059-PD9), Cdk-5 (Roscovitine-Rosc), or p38 MAPK (SB202190-SB2). Inhibitor concentrations are listed in Table 1. After 24 hours of growth factor stimulation, cells were harvested to measure AAH protein by (A) Western blot analysis with (B) digital imaging, or (C) they were analyzed directly in 96-well micro-cultures using the MICE assay. (D) Directional motility was measured using the ALMI assay (see Methods). (A-lower panel) For Western blot controls, the blots were stripped and re-probed with monoclonal antibodies to β-actin. The graphs depict the mean ± S.D. of results. Statistical comparisons were made using ANOVA and post hoc Fisher LSD tests (*P < 0.001 relative to control).

SH-Sy5y cells that were stimulated with IGF-1 for 24 hours in the presence or absence of kinase inhibitor were used to measure AAH protein by Western blot analysis (Figures 5A and 5B) and the MICE assay (Figure 5C). In addition, directional motility was measured using the ALMI assay (Figure 5D). Western blot analysis with the HB-OH monoclonal antibody detected the expected ~86 kD AAH protein in all samples. AAH protein levels were similarly abundant in cells treated with vehicle, SB202190, or H89 (Figures 5A–5B). In contrast, cells treated with Roscovitine, PD98059, Akt inhibitor, or LY294002 had significantly lower levels of AAH protein, and cells treated with LiCl had significantly higher levels of AAH protein relative to control (Figures 5A–5B). Equal loading of protein samples was demonstrated by probing the blots with antibodies to β-actin (Figure 5A). The MICE assay results also demonstrated significantly reduced AAH immunoreactivity in cells treated with the Akt inhibitor, Roscovitine, or PD98059, and increased AAH protein in cells treated with LiCl, which inhibits GSK-3β (Figure 5C). Correspondingly, cells pre-treated with inhibitors of Akt, Erk MAPK, or Cdk-5 had significantly reduced mean total motility indices, while cells pre-treated with LiCl had significantly increased motility (Figure 5D). Pre-treatment with SB202190 had no significant effect on mean total motility relative to control. In essence, the effects of chemical kinase inhibitor treatment on AAH protein levels correlated with their effects on directional motility.

### Cdk-5 Modulation of AAH Expression and Motility

Since the effects of PI3 Kinase/Akt and Erk MAPK have been well documented in relation to growth and motility in various cell types [49-52], we focused further studies to characterize Cdk-5 modulation of AAH, Humbug and Junctin expression as well as directional motility. Cdk-5 activity is increased by the interaction of Cdk-5 protein with one of its regulatory partners, p35 or p25 [26-28]. p35 has a relatively short half-life which may be important for the on-off regulation of Cdk-5 kinase activity, whereas p25, the truncated, C-terminal fragment of p35 [26-28,53-56], has a prolonged half-life and leads to constitutive activation of Cdk-5 kinase [53,55-58]. To examine the effects of Cdk-5 on AAH expression and motility, SH-Sy5y cells were transfected with recombinant plasmid expressing Cdk-5, the p25 or p35 regulatory partner of Cdk-5, Cdk-5+p25, or Cdk-5+p35. Cells transfected with pLuc or empty vector (pcDNA3.1) served as negative controls. In all cases, gene expression was under the control of a CMV promoter. The analyses were performed 48 hours after transfection, corresponding with the peak period of gene expression. For each experiment, the amount of recombinant plasmid and the total quantity of DNA transfected were held constant. To achieve this, empty vector (pcDNA3.1) was used to equalize DNA loading. Cdk-5 activity was measured with in vitro kinase assays using immunoprecipitates and H1 histone as substrate as previously described [58].

**Figure 6:**
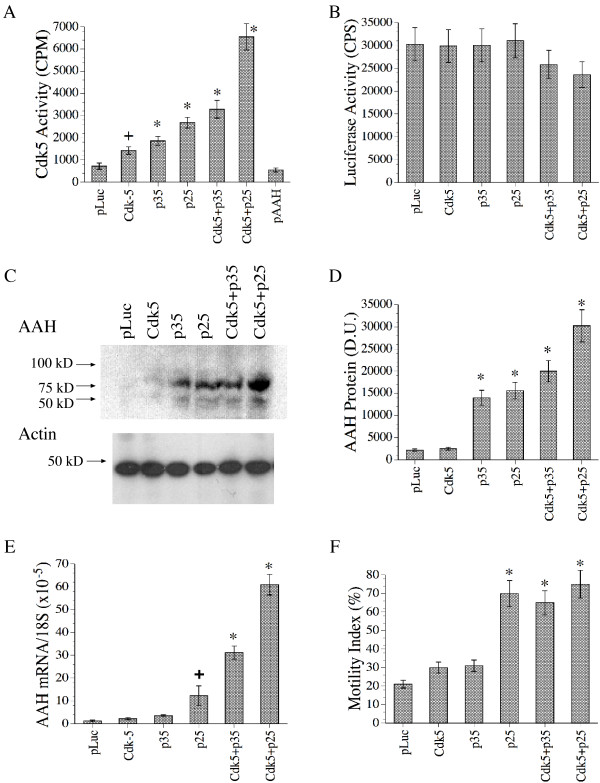
Role of Cdk-5 in the regulation of AAH. SH-Sy5y cells were transfected with recombinant plasmids to over-express Luciferase (pLuc) as a negative control, Cdk-5, p35, p25, Cdk-5+p35, Cdk-5+p25, or pAAH. The cells were harvested 48 hours later to measure (A) Cdk-5 activity, (B) luciferase activity, (C) AAH immunoreactivity by Western blot analysis with b-actin loading control and (D) digital imaging of the Western blot results, (E) AAH mRNA with levels normalized to 18S, and (F) directional motility using the ALMI assay. The graphs depict the mean ± S.D. of results from 6 replicate independent cultures. Inter-group comparisons were made using ANOVA with post hoc Fisher LSD tests (*P < 0.001 and +P < 0.01 relative to pLuc-transfected control cells).

Although cells transfected with Cdk-5, p25, p35, Cdk-5+p25, or Cdk-5+p35 all had significantly increased levels of Cdk-5 activity relative to pLuc-transfected cells, the highest levels of Cdk-5 activity were achieved by transfecting cells with Cdk-5+p25 (Figure 6A). In contrast, cells transfected with pAAH had low levels of Cdk-5 activity, similar to the pLuc-transfected control cells. The transfection efficiencies were similar as demonstrated with a Luciferase reporter assay (Figure 6B). To determine the effects of increased Cdk-5 activity on AAH expression and motility, AAH protein and mRNA levels were measured by Western blot analysis (Figure 6C) with digital image quantification (Figure 6D), and real time quantitative RT-PCR (Figure 6E). Directional motility was measured using the ALMI assay (Figure 6F). Cells transfected with p35, p25, Cdk-5+p35, or Cdk-5+p25 had significantly higher levels of AAH protein expression (Figures 6C and 6D) relative to control cells. In contrast, β-actin expression was similar among the groups (Figure 6C). Cells transfected with p25, Cdk-5+p35, or Cdk-5+p25 also had significantly increased levels of AAH mRNA as demonstrated by real time quantitative RT-PCR (Figure 6E). The directional motility assay results, for the most part, paralleled Cdk-5 activation and AAH expression in that the highest mean directional motility indices were observed in cells transfected with p25, Cdk-5+p35 or Cdk-5+p25 (Figure 6F). In contrast, directional motility was not significantly increased in cells transfected with Cdk-5 or p35 relative to control.

**Figure 7:**
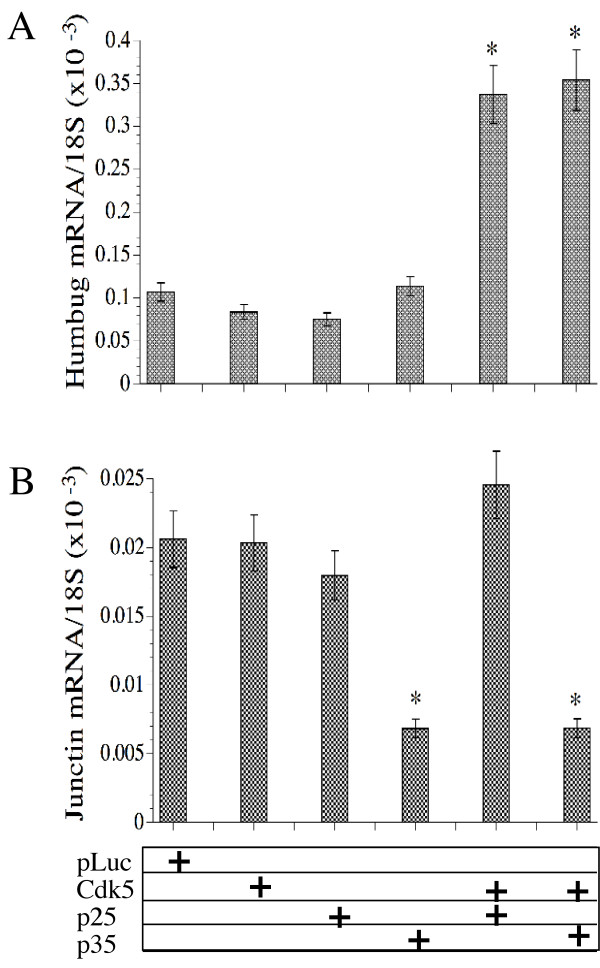
Role of Cdk-5 in the regulation of (A) Humbug and (B) Junctin expression. SH-Sy5y cells were transfected with recombinant plasmids to over-express Luciferase (pLuc) as a negative control, Cdk-5, p25, p35, Cdk-5+p25, or Cdk-5+p35 as indicated in the box below the graphs. The cells were harvested 48 hours later to measure gene expression by real time quantitative RT-PCR (see Methods). The graphs depict the mean ± S.D. of Humbug, or Junction expression with values normalized to 18S rRNA measured in the same samples. Statistical comparisons were made using ANOVA with post hoc Fisher LSD tests (*P < 0.001 relative to pLuc-transfected control cells).

For comparison, we also examined the effects of Cdk-5 activation on Humbug and Junctin expression. Real time quantitative RT-PCR studies demonstrated significantly higher levels of Humbug expression in cells transfected with Cdk-5+p25 or Cdk-5+p35 relative to cells transfected with pLuc, Cdk-5, p25, or p35 (P < 0.001), whereas the cells transfected with Cdk-5+p25 or Cdk-5+p35 had similarly high levels of Humbug (Figure 7A). In contrast, Junctin mRNA levels were not significantly increased in cells transfected with any of the cDNA constructs used, and instead, Junctin mRNA expression was significantly decreased in cells that were transfected with p35 or Cdk-5+p35 (Figure 7B).

### Cdk-5 Mediates Its Effects on AAH and Humbug Expression Through Erk MAPK and PI3-Kinase

**Figure 8:**
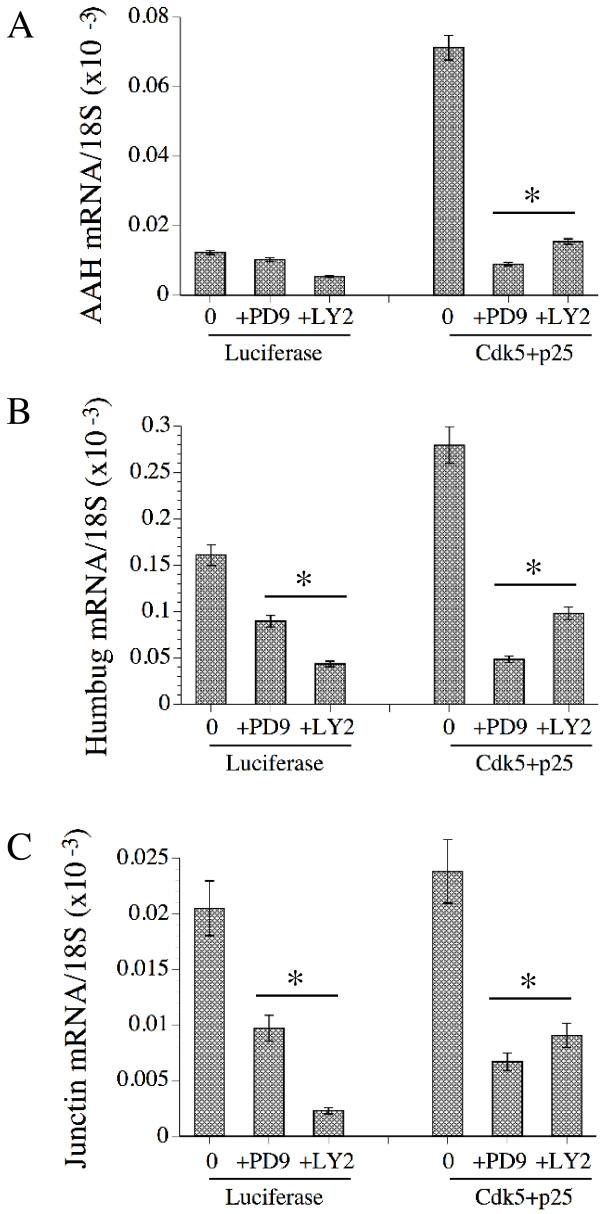
Cdk-5 stimulation of AAH and Humbug expression is mediated by signaling through Erk MAPK and PI3 kinase: SH-Sy5y cells were transfected with recombinant plasmids to over-express Luciferase or Cdk-5+p25. 36 hours after transfection, the cells were treated with vehicle (Con), PD98059 (PD9), or LY294002 (LY2). 12 hours later, i.e. 48 hours after transfection, the cells were harvested to measure (A) AAH, (B) Humbug, or (C) Junctin mRNA levels by real time quantitative RT-PCR. The graphs depict the mean ± S.D. of results normalized to 18S rRNA measured in the same samples. Statistical comparisons were made using ANOVA with post hoc Fisher LSD tests (*P < 0.001 relative to corresponding vehicle-treated control).

To determine if the effects of Cdk-5 on AAH and Humbug expression were mediated downstream through Erk or PI3 kinase, AAH, Humbug, and Junctin mRNA levels were measured in cells transfected with pLuc or Cdk-5+p25. 24 hours prior to harvesting the cells, parallel cultures were treated with PD98059 or LY294002 to inhibit Erk MAPK or PI3 kinase, respectively. Cells transfected with the Cdk-5+p25 had increased AAH and Humbug, but not Junctin mRNA levels by real time quantitative RT-PCR, as described above. However, pre-treatment with PD98059 or LY294002 significantly inhibited AAH, Humbug, and Junctin expression, and with regard to Humbug and Junctin, the inhibition of gene expression occurred independent of Cdk-5+p25 over-expression (Figures 8A–8C).

## Discussion

### Profiles of AAH, Humbug, and Junctin Expression in SH-Sy5y Neuroblastoma Cells and Normal Human Brain

The studies demonstrated expression of all 3 AAH-related mRNA transcripts in SH-Sy5y neuroblastoma cells and CNS-derived PNET cells, with Humbug being the most abundant. PNET2 cells, which are of CNS origin, had similar profiles of AAH, Humbug, and Junctin mRNA transcripts compared with normal infant brains. The significantly higher levels of AAH and Humbug in SH-Sy5y and PNET cells compared with normal brain are consistent with previous reports demonstrating considerably higher levels of AAH and Humbug in transformed compared with non-transformed cells [1,10,12-14,17]. In addition, the higher levels of AAH, Humbug, and Junctin in infant compared with adult human brains suggest that AAH-related molecules are developmentally regulated in the CNS.

### AAH Promotes SH-Sy5y Cell Motility

The structural relationship of Humbug to AAH raised the possibility that Humbug may also promote cell motility. In previous studies, we used antisense oligodeoxynucleotides directed against the 5'end of the AAH mRNA to demonstrate AAH's role in directional motility [14,16]. However, since those reagents would have also inhibited Humbug expression, further studies were needed to determine if AAH alone or together with Humbug mediated cell migration. Herein, we demonstrated that SH-Sy5y cells transfected with pAAH, and not pHMBG, had significantly increased motility relative to pLuc-transfected control cells. Real time RT-PCR studies confirmed that increased motility was associated with increased AAH and not Humbug or Junctin expression. However, these findings did not entirely exclude a role for Humbug since the endogenous expression levels were already high, and further increases may have had a negligible effect. In this regard, Humbug has a demonstrated role in regulating intracellular calcium [5,9,60], and although Humbug may not directly mediate cell motility, its modulation of intracellular pools of calcium may be critical for cytoskeleton reorganization that may be required for cell migration.

### Growth Factor Regulation of AAH, Humbug, and Junctin Expression

Previous observations suggested that AAH expression was modulated by growth factor stimulation. Since AAH, Humbug, and Junctin are transcribed from the same gene, it was of interest to determine the degree to which each of these mRNA transcripts is regulated by growth factor stimulation, particularly insulin and IGF-1. Our focus on insulin and IGF-1 signaling pathways stemmed from earlier studies demonstrating over-expression of AAH in hepatocellular carcinoma cells and in transgenic mice that over-express IRS-1 [23]. The experiments herein demonstrated that AAH and Humbug mRNA's were increased in response to insulin or IGF-1 stimulation, and that Humbug but not AAH expression was increased by NGF stimulation. In addition, the studies showed that Junctin mRNA levels were not significantly modulated by insulin, IGF-1, or NGF. These results indicate that AAH and Humbug expression are transcriptionally regulated by growth factor stimulation, and that since the responses to growth factors are similar but not identical, AAH and Humbug may be differentially regulated by growth factor signaling.

### Downstream Mediators of AAH and Humbug Expression in SH-Sy5y cells

The stimulatory effects of insulin and IGF-1 are mediated by ligand binding and activation of the intrinsic receptor tyrosine kinase [61], which then tyrosyl phosphorylates exogenous cytosolic proteins, including insulin receptor substrate molecules [38,62]. Tyrosyl phosphorylated IRS molecules transmit signals downstream to promote a broad range of functions including growth, survival, energy metabolism, and motility [38,47,62]. The studies herein demonstrated expression of IRS-1, IRS-2, and IRS-4 in SH-Sy5y cells, but substantially higher levels of IRS-1 followed by IRS-4 compared with IRS-2, suggesting that most of the insulin/IGF-1 mediated signaling is transmitted through IRS-1. In the normal human brain, the pattern of IRS expression differed from that observed in SH-Sy5y cells in that the overall levels of IRS gene expression were significantly lower, and IRS-1 was the least abundant while IRS-2 was the most abundant of the three transcripts. This alteration in IRS gene expression, particularly with regard to the up-regulation of IRS-1 in SH-Sy5y cells, is reminiscent of the findings in hepatocellular carcinoma cells [63,64], and suggests that IRS protein levels may be critical for regulating robustness of insulin and IGF-1-transmitted signals, including those that stimulate AAH. In this regard, it is noteworthy that in hepatocellular carcinoma cells, IRS-1 over-expression is associated with increased insulin and IGF-1-stimulated growth and survival signaling, in addition to AAH over-expression relative to the normal liver [11,17,63-65].

Previous studies demonstrated that growth factor stimulated cell motility is mediated by signaling through the Erk MAPK and PI3 kinase-Akt pathways [49-52]. In addition, a probable role for Cdk-5 in relation to neuronal migration during development was demonstrated in mice that were deficient for the Cdk-5 gene [25,28,66-68]. A potential link between insulin and IGF-1 signaling and Cdk-5 activation was suggested by the previous finding that p35 expression was increased by IGF-1 stimulation [69]. Our studies demonstrated prominent IGF-1 stimulation of AAH and Humbug, and significant inhibition of these responses in cells treated with chemical inhibitors of Erk MAPK, Akt, or Cdk-5. These results suggest that IGF-1 stimulated AAH and Humbug expression are signaled through Erk MAPK, Akt, and Cdk-5, and that the effects of these kinases on AAH and Humbug expression are mediated at the level of transcription. The finding that chemical inhibitors of Erk MAPK or PI3 kinase blocked the Cdk-5 stimulated AAH and Humbug expression provides evidence for convergence of these pathways in the regulation of gene expression. Finally, we also observed significantly increased AAH protein but not mRNA expression following LiCl treatment, which inhibits GSK-3β, independent of Akt. The mechanism of this effect is under investigation, but preliminary results suggest that GSK-3β phosphorylation of AAH protein leads to its degradation [Cheng, Tong, and de la Monte, Unpublished].

Previous studies demonstrated a definitive role for growth factor stimulated MAPK mediated cell motility [70]. Erk MAPK signaling can mediate motility of neoplastic cells by activating Rac1 and RhoA GTPases, which promote membrane ruffling, actin-cytoskeletal reorganization, and attendant formation of lamellopodia and filopodia [71]. Similarly, the PI3 kinase/Akt pathway regulates the assembly and re-organization of the actin cytoskeleton [72] and motility [71] by activating Rac1/Cdc42 in response to growth factor stimulation [73]. The downstream effects of Rac1 on cell motility are mediated through Pak1 phosphorylation of LIM kinase [74,75], which phosphorylates targets such as cofilin [76], which in turn promotes actin depolymerization, thereby allowing changes in cell shape and structure. In addition, Rac1 functions through Cdk-5 and p35 to phosphorylate and down-regulate Pak1, which then results in increased neuronal migration [54]. Therefore, growth factor stimulated Rac1 function has an important role in dynamically regulating cytoskeletal reorganization as required for cell migration. Importantly, the convergence of pathways mediating IGF-1 stimulated AAH and Humbug expression may occur through Rac1 and RhoA signaling.

The finding that Cdk-5 activity has a functional role in positively regulating AAH and Humbug expression in SH-Sy5y cells is of particular interest because previous studies provided in vivo evidence that Cdk-5 mediates neuronal migration in the brain during development [28,66]. In this regard, mutant mice lacking either the p35 or Cdk-5 gene have low levels of Cdk-5 activity and exhibit severe defects in neuronal migration [24,28,66,77]. Cdk5 and its regulatory partner, p35 or p39, have also been implicated in growth cone motility during axon extension [25,27,78]. One mechanism of Cdk-5-mediated neuronal migration involves interactions between Cdk5-p35 and Rac GTPase, which is required for growth cone motility [25,54]. Cdk-5 can mediate its effects on neuronal migration through POU domain-containing transcription factors such as Brn 1 and Brn 2, which have roles in neuronal migration [79] or MEF-2, which is a target of Cdk-5-mediated phosphorylation [80], and has a known role in cellular differentiation [81,82]. Although Cdk-5-mediated neuronal migration has been studied mainly in post-mitotic neurons, the present work demonstrates that Cdk-5 signaling is also relevant to immature proliferating and transformed neuronal cells. Our results demonstrating that increased Cdk-5 activity results in increased AAH and Humbug expression are consistent with the concept that Cdk-5 mediates neuronal migration [24,28,66,77], and further suggest that AAH and Humbug are downstream targets of Cdk-5 signaling.

## Conclusion

AAH and Humbug are over-expressed in SH-Sy5y neuroblastoma cells, and their mRNAs are regulated by insulin/IGF-1 signaling through Erk MAPK, PI3 kinase-Akt, and Cdk-5, which are known mediators of cell migration. Although AAH and Humbug share regulatory signaling pathways, AAH and not Humbug mediates directional motility in SH-Sy5y neuroblastoma cells.

## Abbreviations

AAH: aspartyl-(asparaginyl)-β-hydroxylase

ALMI assay: ATP luminescence-based motility/invasion assay

ANOVA: analysis of variance

BSA: bovine serum albumin

Cdk-5: cyclin-dependent kinase 5

CMV: cytomegalovirus

CNS: central nervous system

DMEM: Dulbecco's modified Eagle's medium

EDTA: ethylenediaminetetraacetic acid

EGF: epidermal growth factor

EGTA: ethylene glycol-bis(2-aminoethyl ether)-N,N,N',N'-tetraacetic acid

ELISA: enzyme-linked immunosorbant assay

Erk MAPK: extracellular signal-regulated kinase, mitogen-activated protein kinase

GSK: glycogen synthase kinase

IGF: insulin-like growth factor

IRS: insulin-receptor substrate

Fisher LSD: Fisher least significant difference

MICE: microtiter immunocytochemical ELISA

NGF: nerve growth factor

pAAH: AAH-expressing plasmid

pHMBG: Humbug-expressing plasmid

PI3 kinase: phosphatidylinositol 3-kinase

PKA: protein kinase A

pLuc: Luciferase-expressing plasmid

PMSF: phenylmethylsulphonylfluoride

PNET: primitive neuroectodermal tumor

RT-PCR: reverse transcriptase polymerase chain reaction

SDS-PAGE: sodium dodecyl sulfate-polyacrylamide gel electrophoresis

TBS: Tris buffered saline

TBST: Tris buffered saline with 0.05% Tween-20

TPCK: tosylphenylalanylchloromethane

## Competing interests

The author(s) declare that they have no competing interests.

## Authors' contributions

SAL participated in all phases of the research and in drafting the manuscript. JJC contributed by performing Western blot analysis, cell culture stimulation assays, and cellular ELISAs. XJX contributed by performing kinase assays and Western blot analysis. JRW had a role in conceptualizing the experiments and critically revising the manuscript. SMdlM had a major role in the conceptualization and design of the experiments, analyzing results and was chiefly responsible for drafting and critically revising the manuscript. All authors read and approved the final manuscript.
